# PathCNN: interpretable convolutional neural networks for survival prediction and pathway analysis applied to glioblastoma

**DOI:** 10.1093/bioinformatics/btab285

**Published:** 2021-07-12

**Authors:** Jung Hun Oh, Wookjin Choi, Euiseong Ko, Mingon Kang, Allen Tannenbaum, Joseph O Deasy

**Affiliations:** Department of Medical Physics, Memorial Sloan Kettering Cancer Center, New York, NY 10065, USA; Department of Computer Science, Virginia State University, Petersburg, VA 23806, USA; Department of Computer Science, University of Nevada, Las Vegas, NV 89154, USA; Department of Computer Science, University of Nevada, Las Vegas, NV 89154, USA; Departments of Computer Science and Applied Mathematics & Statistics, Stony Brook University, New York, NY 11794, USA; Department of Medical Physics, Memorial Sloan Kettering Cancer Center, New York, NY 10065, USA

## Abstract

**Motivation:**

Convolutional neural networks (CNNs) have achieved great success in the areas of image processing and computer vision, handling grid-structured inputs and efficiently capturing local dependencies through multiple levels of abstraction. However, a lack of interpretability remains a key barrier to the adoption of deep neural networks, particularly in predictive modeling of disease outcomes. Moreover, because biological array data are generally represented in a non-grid structured format, CNNs cannot be applied directly.

**Results:**

To address these issues, we propose a novel method, called *PathCNN*, that constructs an interpretable CNN model on integrated multi-omics data using a newly defined *pathway image*. PathCNN showed promising predictive performance in differentiating between long-term survival (LTS) and non-LTS when applied to glioblastoma multiforme (GBM). The adoption of a visualization tool coupled with statistical analysis enabled the identification of plausible pathways associated with survival in GBM. In summary, PathCNN demonstrates that CNNs can be effectively applied to multi-omics data in an interpretable manner, resulting in promising predictive power while identifying key biological correlates of disease.

**Availability and implementation:**

The source code is freely available at: https://github.com/mskspi/PathCNN.

## 1 Introduction

Deep neural network methods provide the capability to perform non-linear modeling, while handling complex structures and dependencies in data, in order to learn informative representations through multiple levels of abstraction (LeCun *et al.*, 2015; Lin *et al.*, 2020). In particular, the convolutional neural network (CNN) has achieved great success in the field of image processing and recognition, dealing with grid-structured inputs or images and efficiently capturing local dependencies between neighboring pixels (Cheng *et al.*, 2016; Yamashita *et al.*, 2018; Zhang *et al.*, 2019). There have been several studies applying CNNs to bioinformatics problems (Min *et al.*, 2017), for example, in learning protein–RNA binding preferences (Ben-Bassat *et al.*, 2018), predicting the sequence specificities of DNA/RNA-binding proteins (Alipanahi *et al.*, 2015), and identifying the functional effects of non-coding variants (Zhou and Troyanskaya, 2015). On the other hand, it appears that traditional neural networks rather than CNNs have been extensively employed to solve biological problems due to the non-grid structured format typically represented in biological array data. Recently, multiple studies have utilized traditional neural networks on multi-omics data provided by The Cancer Genome Atlas (TCGA) project for the analysis of various molecular components in cancer genetics (Spainhour *et al.*, 2019), including the identification of cancer subtypes (Mallavarapu *et al.*, 2020) and prediction of cancer treatment survival (Hao *et al.*, 2019a,b). Comparatively, however, few studies have adopted CNNs for multi-omics data analysis.

The lack of interpretability is a crucial factor that limits the adoption of neural networks in the field of medicine, where biological interpretation of the trained models or results is particularly important to better understand the biological mechanisms of complex human diseases (Vamathevan *et al.*, 2019). To increase the interpretability of classical CNNs in image problems, an approach called *Class Activation Mapping* (CAM) was developed using global average pooling (Zhou *et al.*, 2016). CAM produces a localization map for a target class, visualizing the discriminative image regions used by the CNN to predict the class. However, CAM has some limitations: (i**)** to employ CAM, the CNN architecture used in modeling should be modified by removing fully connected layers and adding a global average pooling layer after the last convolutional layer where class activation maps are generated, and (ii**)** the modified network for CAM should be fine-tuned. A more generalizable approach, called *Gradient-weighted Class Activation Mapping* (Grad-CAM), was subsequently proposed, which uses the gradient information of any target class flowing into the last convolutional layer to produce class activation maps (Selvaraju *et al.*, 2017).

In this study, we propose a novel method, called *PathCNN*, to build an interpretable CNN model of cancer outcomes using multi-omics data. As input data to the CNN model, pseudo images of biological pathways (called *pathway images*) are used, which are generated in the low dimensional space of integrated multi-omics data including mRNA expression, copy number variation (CNV) and DNA methylation. After modeling, to identify the biological pathways associated with outcomes, Grad-CAM is used, for which attention maps superimposed on pathway images are analyzed to pinpoint key pathways.

We demonstrate the method applied to predicting long-term survival in patients diagnosed and treated for glioblastoma multiforme (GBM). GBM is one of the most malignant tumors with poor prognosis and outcomes with a median survival of 14–15 months after diagnosis (Hanif *et al.*, 2017; Hou *et al.*, 2006). The identification of key biomarkers associated with survival in GBM patients could further help elucidate the underlying biological mechanisms that play a role in the biology of GBM. Through the proposed methods, we show that the resulting CNN model achieves much better predictive performance compared to other methods. Furthermore, the model employing Grad-CAM is interpretable, enabling the visual identification of influential biological pathways.

## 2 Materials and methods

### 2.1 Data

Multi-omics data for GBM, including mRNA expression, CNV and DNA methylation, denoted as *G*, *C* and $M\in R^{n\times r}$, respectively, were downloaded from the cBioPortal database (Cerami *et al.*, 2012), where *n* and *r* indicate the numbers of samples and genes, respectively. Long-term survival (LTS) was defined as survival > 2 years after diagnosis, whereas non-LTS was defined as survival ≤ 2 years. Individuals who survived with the last follow-up ≤ 2 years were excluded in further analysis.

### 2.2 Pathway image

**Fig. 1.**
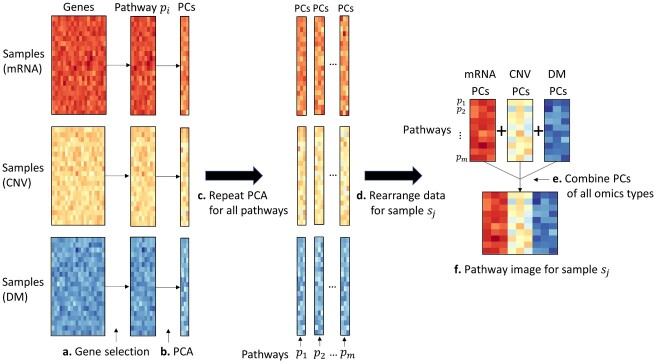
Generation ofpathway images from multi-omics data using principal component analysis (PCA). PCA was carried out for individual pathways with associated genes in each omics type. A pathway image for a sample was produced by combining a few principal components of all omics types. PCA, principal component analysis; CNV, copy number variation; DM, DNA methylation

Each type of omics data at a gene level was converted into pathway level profiles. To do this, pathway information was first extracted, along with the associated genes for each pathway from the Kyoto Encyclopedia of Genes and Genomes (KEGG) database (Kanehisa *et al.*, 2016). After excluding disease-specific pathways, 146 pathways were used. For a pathway *p_i_*, the mRNA expression data of associated genes were extracted from the mRNA expression matrix (*G*), producing an intermediate matrix $B\in R^{n\times r_{i}}$, where *r_i_* is the number of genes involved in the pathway *p_i_*. That is, the matrix *B* consists of samples in rows and genes for a given pathway in columns. Using principal component analysis (PCA), the matrix *B* was decomposed into uncorrelated components, yielding $G_{p_{i}}\in R^{n\times q}$, where *q* is the number of principal components (PCs). This task for the pathway *p_i_* was also carried out on the CNV matrix (*C*) and DNA methylation matrix (*M*), yielding $C_{p_{i}},\;M_{p_{i}}\in R^{n\times q}$, respectively. The process was repeated for all 146 pathways, resulting in the merged matrices *G_p_*, *C_p_* and $M_{p}\in R^{n\times 146q}$ for the 146 pathways. Lastly, by rearranging the matrices for each sample *s_j_*, a set of matrices, $G_{s_{j}},\;C_{s_{j}},\;M_{s_{j}}\in R^{146\times q}$, was produced. A combined matrix of the three matrices, $K_{s_{j}}\in R^{146\times 3q}$, is called the *pathway image* of the sample *s_j_*, where rows represent 146 pathways, and columns represent 3×q PCs combined for the three omics types, which was input into a CNN model. In this study, a few PCs (*q*** **=** **1–5) were used; for example, a sample (pathway image) is represented by a matrix with 146 × 6 elements for *q*** **=** **2, where the first and second columns are from mRNA expression, the third and fourth columns are from CNV, and the fifth and sixth columns are from DNA methylation, representing the first two PCs of each omics type. Figure 1 illustrates the process to generate the pathway images.

### 2.3 Order of pathways

To identify the important pathways associated with LTS in GBM, Grad-CAM was used, which is a technique to localize discriminative image regions. Thus, if correlated pathways are clustered on pathway images, Grad-CAM is more likely to identify key pathways. This is also consistent with the nature of CNNs that capture local patterns in input images through various filters. It is intuitively reasonable to place correlated pathways in proximity on the pathway images to better localize important regions. Hence, the 146 pathways were ordered in the following manner: Pearson correlation between pathways was computed on a matrix of 146 by (number of samples × number of PCs × 3), generated by combining all resultant pathway images. The two most correlated pathways were placed on the top two rows on the pathway images, and then a pathway that was the most correlated with the pathway in the second row was placed on the third row. This process was repeated for all 146 pathways. Note that the *i*th pathway was located among those not previously selected, and it was the most correlated with the pathway in the row *i*−1. As a result, all pathway images had the same order of pathways.

### 2.4 CNN architecture

**Fig. 2.**
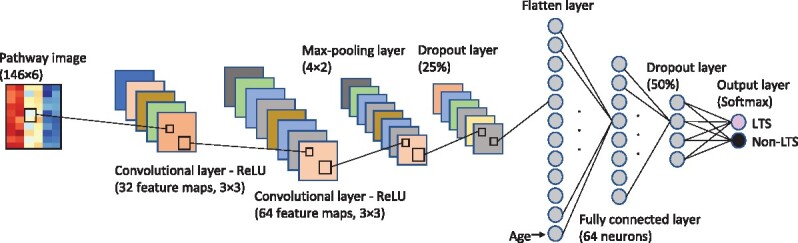
The convolutional neural network architecture used in this study for long-term survival prediction in glioblastoma. The input layer was provided with pathway images. A clinical variable, age, was connected to the fully connected layer. LTS, long-term survival; Non-LTS, non-long-term survival

The input layer was provided with pathway images. The CNN architecture consisted of two convolutional layers with 32 and 64 filters, with a size of 3 × 3, followed by a 4 × 2 max-pooling layer, and a dropout layer with a dropout rate of 25%. Each convolutional layer was immediately followed by a rectified linear unit (ReLU) activation function (Nair and Hinton, 2010). The output from the dropout layer was flattened to a 1D vector and connected to a fully connected layer of 64 nodes, followed by a dropout layer with a dropout rate of 50%, and a softmax layer. A clinical variable, age, was connected to the fully connected layer. Figure 2 illustrates the CNN architecture. The training epoch was set to 30, and the batch size was set to 64 for all runs. Adam was used as an optimizer with a learning rate of 0.0001. We used Keras 2.3 with Tensorflow 2.0 as the backend; the code was run in Google Colab using GPUs.

### 2.5 Cross validation

Modeling used a 5-fold cross validation (CV) approach with 30 repeats. In each 5-fold CV, samples were randomly assigned as follows: 20% of data were assigned as a test set for performance evaluation, and the remaining data were further split into a training set (80% of the remaining data) and a so-called validation set (20% of the remaining data) used to tune hyper-parameters. The modeling was carried out using the training set and optimizing hyper-parameters in the CNN model using the validation set. The model was built in each CV and was evaluated on the test set. Predictive performance was quantified by the area under the curve (AUC).

### 2.6 Biological interpretation using Grad-CAM

**Fig. 3.**
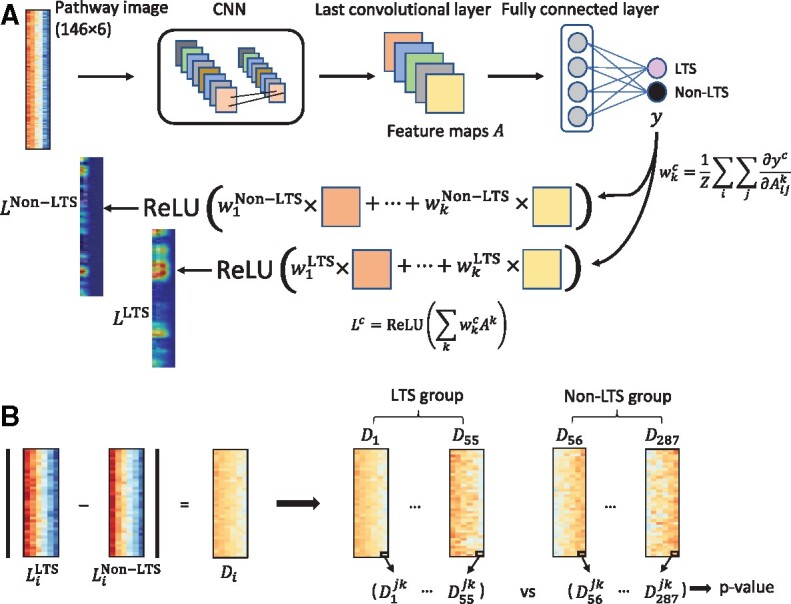
An illustration of biological interpretation. (**A**) Grad-CAM procedure to generate class activation maps. The two images on the left bottom represent an example of the class activation maps for a sample in the cohort, which were generated from Grad-CAM procedure; (**B**) statistical analysis to identify significantly different pathways between the LTS and non-LTS groups. LTS, long-term survival; CNN, convolutional neural network; ReLU, rectified linear unit

Grad-CAM for class-discriminative localization mapping was employed to identify the important pixels (pathways) on pathway images associated with LTS in GBM patients (Fig. 3A) (Selvaraju *et al.*, 2017). Class activation maps were generated by computing the gradient of a score ($y^{c}$) for each class *c* with respect to feature maps *A* of the last convolutional layer. More specifically, neuron importance weights $w^{c}$ for a class *c* were computed as follows:

(1) $$w_{k}^{c}=\frac{1}{Z}\sum_{i}\sum_{j}\frac{\partial y^{c}}{\partial A_{ij}^{k}}$$

where *k* is the number of feature maps and *Z* is the number of pixels in the feature map. An activation map for the class *c* was then produced using the following equation:

(2) $$L^{c}=\text{ReLU}\left(\sum_{k}w_{k}^{c}A^{k}\right)$$

where *L^c^* is a weighted sum of feature maps followed by a ReLU. The motivation of the ReLU is to highlight pixels whose intensities contribute to increasing $y^{c}$, having a positive influence on the target class. A localization map for each class was normalized to lie between 0 and 1. Since the class activation map has the same size as the feature maps, it was up-sampled to the size of the input pathway image.

Unlike the modeling with cross validation, for biological interpretation using Grad-CAM, a CNN model was built using all samples. Each sample was fed into the model, leading to two activation maps (for LTS and non-LTS). A difference map between the two activation maps was thereby produced. After repeating this process for all samples, a statistical analysis was conducted. For a given pixel, the statistical difference between the LTS and non-LTS groups was assessed using the Wilcoxon rank-sum test (Fig. 3B). The *P*-values were corrected for multiple testing using the Bonferroni correction.

### 2.7 Comparison with benchmark methods

The predictive performance of PathCNN was compared with several machine learning methods, including logistic regression, support vector machines (SVMs), fully connected neural networks and the recently proposed Multi-omics Integrative Net (MiNet) (Hao *et al.*, 2019a). For all experiments, the same experimental settings were used as described in the above Cross validation section. For SVM, several kernels, including radial basis function (RBF), polynomial and sigmoid kernels were assessed. For the fully connected neural network, a neural network architecture was empirically tuned, which consisted of an input layer, five hidden layers and an output layer. The numbers of nodes in the hidden layers were 10k, 7.5k, 5k, 2.5k and 500, respectively. ReLU was used as an activation function between the hidden layers and a sigmoid function in the output layer. The rate of dropout in the hidden layers was set to 80%. The architecture of MiNet, originally designed to compute a concordance index, was modified, by replacing the loss function of negative log partial likelihood with binary cross entropy to compute an AUC. Both the neural network and MiNet models were trained by the Adam optimizer. For all experiments, the multi-omics data and a clinical variable of age were input to each machine learning model.

## 3 Results

### 3.1 Data

The TCGA multi-omics data of GBM consisted of mRNA expression, CNV and DNA methylation, measured at the gene level: mRNA expression for 12 042 genes in 528 cases, CNV for 24 776 genes in 577 cases and DNA methylation for 11 807 genes. For DNA methylation data, two Illumina Infinium DNA methylation bead arrays (HM27 and HM450) were used for 285 and 155 cases, respectively. Removing 5 duplicate cases, 435 cases were evaluable for DNA methylation data. In total, 8037 genes were common in the three omics types, and 343 cases had all three omics types. After removing individuals who survived with the last follow-up ≤ 2 years, LTS and non-LTS groups had 55 and 232 cases, respectively. To handle imbalanced data, class weights were set in the modeling according to the ratio of the number of samples between the two groups.

In total, 4989 unique genes were involved in 146 KEGG pathways with an average of 68 genes per pathway. PCA tests were performed for individual pathways separately on each omics type to convert gene level information to pathway level information. When genes belonging to a given pathway were not available, PCA was conducted without the missing genes. The number of missing genes in each pathway was on average 13, 2 and 20 for mRNA expression, CNV and DNA methylation, respectively.

The average age in LTS and non-LTS groups was 48 and 61 years, respectively, and the difference in age between the two groups was statistically significant with a *P*-value < 0.001 using a two-sample t-test. Due to the significance of age in terms of survival, the variable was added to the fully connected layer in the CNN model. IDH-wildtype GBM is more common and has a worse prognosis, whereas IDH-mutant GBM is more frequently observed in younger patients and has been associated with longer survival (Burgenske *et al.*, 2019). There were IDH mutations in only 6 and 2 cases in the LTS and non-LTS groups, respectively; however, the difference was statistically significant with Fisher’s exact test *P* = 0.0013.

### 3.2 Modeling performance

**Fig. 4.**
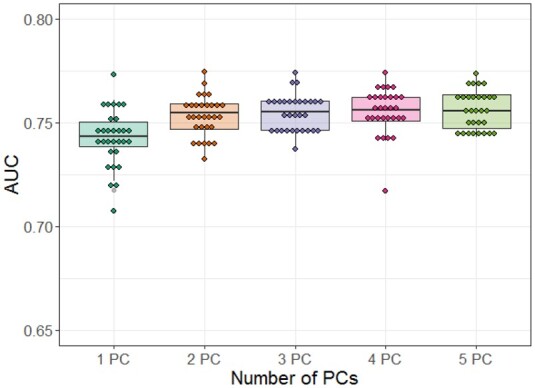
Performance comparison of convolutional neural network models. The different size of pathway image was assessed with the top one principal component through five principal components in the generation of pathway images. PC, principal component; AUC, area under the curve

The CNN model trained to classify LTS and non-LTS groups in GBM using pathway images was tested in a 5-fold CV scheme. The pathway image representation consisted of 146 rows (each row represents the same pathway) and 3×q columns, where *q* denotes the number of PCs. For example, for *q*** **=** **2, the columns were formed with the first two PCs for each omics type in the following order: mRNA expression, CNV and DNA methylation. In the modeling, various sizes of pathway images were assessed with *q*** **=** **1 through 5. In each experiment, an average AUC over 30 iterations of the 5-fold CV was reported. As shown in Figure 4, when two PCs (*q*** **=** **2) were used, the performance was saturated with an average AUC of 0.753, and there was no significant additional improvement with more PCs. When *q*** **=** **2, a model without age achieved an average AUC of 0.677, demonstrating the significance of age.

All possible arrangement combinations of mRNA expression, CNV and DNA methylation in pathway images with two PCs were tested. Interestingly, an arrangement of (CNV, mRNA expression and DNA methylation) resulted in the worst AUC of 0.736. Arrangements of (CNV, DNA methylation and mRNA expression) and (DNA methylation, mRNA expression and CNV) achieved average AUCs of 0.741 and 0.747, respectively. Other arrangements resulted in average AUCs of 0.755 which is similar to that of the arrangement order of (mRNA expression, CNV and DNA methylation) used in this study. The performance differences, though small, may be due to the relatedness of the different data types.

**Fig. 5.**
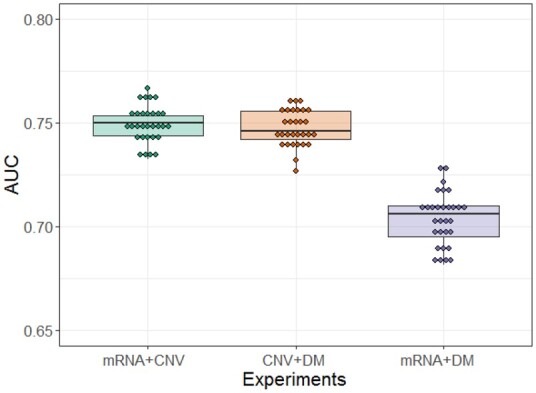
Performance comparison of convolutional neural network models with a combination of two omics types. AUC, area under the curve; CNV, copy number variation; DM, DNA methylation

To assess which omics type is more informative to the model, CNN modeling on pathway images, generated using combinations of two omics types, was conducted with *q*** **=** **2. As shown in Figure 5, a combination of mRNA expression and CNV, and a combination of CNV and DNA methylation, achieved comparable performance with average AUCs of 0.749 and 0.748, respectively. A combination of mRNA expression and DNA methylation showed much worse performance with an average AUC of 0.704. In addition, the CNN modeling on pathway images with a single omics type showed average AUCs of 0.699, 0.715 and 0.687 for mRNA expression, CNV and DNA methylation, respectively. Here, *q*** **=** **3 was used due to the filter size of 3 × 3. Overall, the use of multi-omics types in modeling showed improved performance. However, DNA methylation did not significantly improve the predictive power compared to mRNA expression and CNV, which is likely due to the relatively high rate of missing genes in DNA methylation. By contrast, the low missing rate in CNV explains its contribution in predictive power.

### 3.3 Comparison with benchmark methods

The predictive performance of PathCNN was compared with four different machine learning methods, including logistic regression, SVMs, fully connected neural networks and MiNet. In addition to GBM, multi-omics data, including RNASeq gene expression, CNV and DNA methylation, were downloaded from the TCGA and assessed for three other cancers, including kidney cancer, low-grade glioma (LGG) and lung adenocarcinoma (LUAD). Table 1 lists the number of evaluable genes in each cancer. Because these three cancers are relatively less malignant compared to GBM, a threshold of 3 years was used to define LTS. That is, LTS was defined as survival > 3 years. After removing individuals who survived but had their last follow-up ≤ 3 years, the LTS and non-LTS groups had 154 and 69, 156 and 75 and 110 and 115 cases for kidney cancer, LGG and LUAD, respectively. Class weights were set in the modeling according to the ratio of the number of samples between the two groups. For LGG, age was also input to each machine learning model. Modeling was not conducted with the threshold of 3 years for GBM, due to worse survival and the resulting extreme class imbalance (LTS and non-LTS groups at 3 years had 23 and 256 cases, respectively.) In each experiment, an average AUC over 30 iterations of the 5-fold CV was reported. As shown in Table 2, for most cancers, PathCNN outperformed other machine learning modeling methods. However, in LGG, SVM with RBF had slightly better performance than PathCNN. It should be noted that in all experiments, PathCNN had the smallest standard deviation, implying an increased stability compared with other approaches.

**Table 1.** The number of genes in each omics type

Cancer type	RNASeq expression	CNV	DNA methylation
Low-grade glioma	20 440	24 776	16 603
Lung adenocarcinom	20 440	24 776	16 565
Kidney cancer	20 440	24 776	16 459

CNV, copy number variation.

**Table 2.** Comparison of predictive performance with benchmark methods in terms of the area under the curve (AUC: mean ± standard deviation) over 30 iterations of the 5-fold cross validation

Cancer	PathCNN	Logistic regression	SVM with RBF	Neural network	MiNet
GBM	**0.755 **±** 0.009**	0.668 ± 0.039	0.685 ± 0.037	0.692 ± 0.030	0.690 ± 0.032
LGG	0.877 ± 0.007	0.816 ± 0.036	**0.884 **±** 0.017**	0.791 ± 0.031	0.854 ± 0.027
LUAD	**0.637 **±** 0.014**	0.581 ± 0.028	0.624 ± 0.034	0.573 ± 0.031	0.597 ± 0.042
KIRC	**0.709 **±** 0.009**	0.654 ± 0.034	0.684 ± 0.027	0.702 ± 0.028	0.659 ± 0.030

*Note*: AUCs for PathCNN were obtained with three principal components. Bold = Highest AUC for each dataset.

SVM, support vector machine; RBF, radial basis function; MiNet, Multi-omics Integrative Net; GBM, glioblastoma multiforme; LGG, low-grade glioma; LUAD, lung adenocarcinoma; KIRC, kidney cancer.

### 3.4 Identification of key pathways

To assist biological interpretations, the CNN model was retrained using all samples of the pathway images generated with the first two PCs for individual omics types. To identify the biological mechanisms associated with survival in GBM patients and independent from age, the clinical variable age was removed from the CNN model. Individual pathway images were fed into the trained model, as shown in Figure 3A. Grad-CAM then generated two activation maps (LTS and non-LTS) for each sample in the last convolutional layer. Let $L_{i}^{\text{LTS}}$ and $L_{i}^{\text{non}-\text{LTS}}$ denote the two activation maps for LTS and non-LTS for a sample *s_i_*, respectively. If the model is well trained, and an input sample *s_i_* belongs to the LTS group, $L_{i}^{\text{LTS}}$ is more likely to be activated than $L_{i}^{\text{non}-\text{LTS}}$, and vice-versa. After up-sampling the two activation maps to the original input size, the absolute intensity differences of individual matched pixels between the two activation maps were computed in a normalized range, i.e. $D_{i}=|L_{i}^{\text{LTS}}-L_{i}^{\text{non}-\text{LTS}}|$. After the Grad-CAM procedure for all samples, a set of matrices $D=(D_{1},D_{2},\ldots ,D_{n})$ was produced, where *n* is the number of samples. Based on the original class of each sample, *D* was split into two groups, $D^{\text{LTS}}$ and $D^{\text{non}-\text{LTS}}$. For a pixel with an index (*j*, *k*), i.e. *j*th row and *k*th column on each matrix in the set *D*, the difference in values between $D^{\text{LTS}}$ and $D^{\text{non}-\text{LTS}}$ was assessed using a Wilcoxon rank-sum test, yielding a *P*-value. After statistical tests for all pixels, *P*-values were corrected using the Bonferroni correction.

**Fig. 6.**
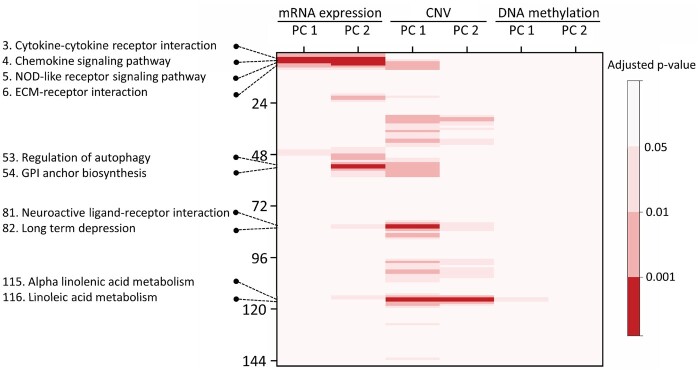
A matrix of adjusted *P*-values. The row represents the 146 KEGG pathways ordered on pathway images. The columns represent the first two principal components of each omics type. The red color indicates key pathways with adjusted *P*-values < 0.001

**Fig. 7.**
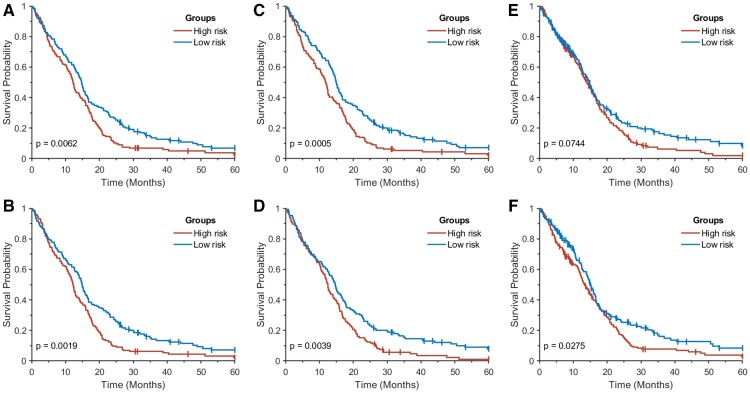
Kaplan–Meier curves for two groups dichotomized by a median split in PC1 of mRNA expression: (**A**) cytokine-cytokine receptor interaction; (**B**) chemokine signaling pathway; (**C**) NOD-like receptor signaling pathway; and (**D**) ECM–receptor interaction, and Kaplan–Meier curves dichotomized by a median split in PC1 of CNV: (**E**) neuroactive ligand-receptor interaction; and (**F**) linoleic acid metabolism

As a result of the statistical tests, four activation areas with adjusted *P*-values < 0.001 were found, which consisted of 15 pixels (pathways) with 10 unique pathways (Fig. 6, Table 3). Note that each pixel indicates a PC. All 15 pixels in the four hot spots were found in PCs of either mRNA expression or CNV. Three pathways, including the cytokine-cytokine receptor interaction, chemokine signaling pathway and NOD-like receptor signaling pathway, were enriched in both PC 1 and PC 2 for mRNA expression. Two pathways, including the alpha linolenic acid metabolism and linoleic acid metabolism, were enriched in both PC 1 and PC 2 for CNV. Clearly, the linoleic acid metabolism and alpha linolenic acid metabolism are highly correlated, consisting of 29 and 19 genes, respectively, with 16 common genes. A Kaplan-Meier analysis was performed to compare survival time between two groups dichotomized by a median split of PC values in the key pathways. That is, given a key pathway (pixel), PC values across all samples were dichotomized by a median split. Figure 7 shows the results for four pathways, including the cytokine-cytokine receptor interaction, chemokine signaling pathway, NOD-like receptor signaling pathway and ECM-receptor interaction, in PC1 of mRNA expression. All were statistically significant on log-rank tests. Kaplan-Meier curves dichotomized by a median split of PC1 values in CNV are shown in Figure 7E and F. The linoleic acid metabolism had statistical significance with a log-rank *P*-value of 0.0275. The neuroactive ligand-receptor interaction had borderline significance with a log-rank *P*-value of 0.0744 whereas other pathways were not statistically significant.

**Table 3.** Key pathways associated with the long-term survival in GBM patients

Row	Pathway	Gene expression		CNV	
		PC 1	PC 2	PC 1	PC 2
3	Cytokine-cytokine receptor interaction	0.0003	0.0008		
4	Chemokine signaling pathway	0.0001	0.0001		
5	NOD-like receptor signaling pathway	0.0003	0.0001		
6	ECM-receptor interaction		0.0006		
53	Regulation of autophagy		0.0006		
54	Glycosylphosphatidylinositol (GPI) anchor biosynthesis		0.0005		
81	Neuroactive ligand-receptor interaction			0.0005	
82	Long term depression			0.0008	
115	Alpha linolenic acid metabolism			0.0001	0.0002
116	Linoleic acid metabolism			0.0008	0.0007

*Note*: The column Row indicates the row numbers of pathways on pathway images.

PC, principal component; CNV, copy number variation.

## 4 Discussion

GBM is one of the most aggressive and malignant forms of cancer, with mostly poor outcomes (Hanif *et al.*, 2017; Hou *et al.*, 2006). Although GBM is rare with an annual incidence of <10 per 100 000 people, a poor median survival of 14–15 months after diagnosis makes it a major public health concern (Chou *et al.*, 2018; Hatoum *et al.*, 2019). The identification of key biomarkers that play a role in the biology of GBM could help better understand the underlying biological mechanisms of GBM aggressiveness. Many studies have reported putative survival-associated biomarkers in GBM using a single-omics platform (Dong *et al.*, 2019; Karsy *et al.*, 2015). However, the analysis of data from individual assays currently lacks the capability of finding robust biomarkers that are able to guide therapeutic decisions in GBM (Xiong *et al.*, 2014). An integrated analysis of multi-omics data can potentially provide useful insights into the complex biology and molecular heterogeneity of cancer, taking into account the interactions of different types of biomarkers (Montaner *et al.*, 2020).

In recent years, CNNs have been successfully applied to computer vision and image processing problems, achieving state-of-the-art performance (Cheng *et al.*, 2016; Li *et al.*, 2018). However, there is a limitation of applying CNNs to bioinformatics problems, since in general, biological data are represented as non-grid structures. Another limitation of using deep learning techniques on biological data lies in the interpretability of the trained model and findings, often treated as a ‘black box’. The interpretation of deep neural network models is particularly important in biological data analysis to derive the underlying biology of diseases.

To adapt CNNs to biological pathway analysis, we have proposed using a new formulation of ‘pathway images’ to represent multi-omics data. To leverage CNN capabilities, pathways with greater correlated activity were arranged more closely in the pathway image. Use of principle components effectively summarizes correlated pathway activity. The resulting CNN modeling on the integrated multi-omics data at the pathway level improved predictive power, adding complementary information to the model. It is likely that such an analysis at the pathway level removes noisy information that may exist in individual omics types, representing each pathway as a linear combination of relevant genes in the principal component space. The adoption of Grad-CAM after the CNN modeling effectively enabled biological interpretations, and thus, identifying pathways likely associated with survival in GBM patients. Non-linear dimensional reduction techniques, such as t-distributed stochastic neighbor embedding (t-SNE) and uniform manifold approximation and projection (UMAP), will be further considered in future work.

In the 146 pathways, the number of genes involved in each pathway ranged from 10 (taurine and hypotaurine metabolism) to 389 (olfactory transduction). For the sake of simplicity and efficiency in CNN modeling and Grad-CAM based biological interpretation, the size of the pathway image for each sample was fixed with 146 pathways in rows and a predefined number of PCs in columns. For the dimensional reduction of each pathway, if there were missing genes in the omics data, PCA was performed without those genes, which removed the difficulty of handling missing data. Despite the linear nature of PCA, the non-linear relationships among pathways can be captured by the CNN model.

The resulting pathway based CNN model effectively predicted LTS for GBM patients. When all three omics types were used, an average AUC of 0.753 was obtained, using the first two PCs for each omics type. When two omics types (mRNA expression with CNV and CNV with DNA methylation) were used, similar performance was achieved with average AUCs of 0.749 and 0.748, respectively. Predictive power worsened when mRNA expression with DNA methylation data were used, with an average AUC of 0.704. Overall, the use of multi-omics data significantly improved predictive performance compared to modeling using any single omics type.

For the biological interpretation of the class activation maps that were derived using a Grad-CAM technique, a matrix was produced that represents the intensity difference between the two class activation maps for each sample. Then, for a specific pixel on the difference maps, a statistical analysis was conducted to assess the value differences between the LTS and non-LTS groups. Before that, a normality test, using the Kolmogorov-Smirnov method, was performed. For all pixels, the Kolmogorov-Smirnov test produced *P*-values < 0.05, indicating that all tests rejected the null hypothesis that the data came from a normal distribution at the 5% significance level. Therefore, non-parametric tests with a Wilcoxon rank-sum method were carried out.

Using the Grad-CAM, four significant regions with adjusted *P*-values < 0.0001 were found, in which at least two correlated pathways were localized together, consisting of 15 pixels and 10 unique pathways: (i) cytokine-cytokine receptor interaction, chemokine signaling pathway, NOD-like receptor signaling pathway, ECM-receptor interaction; (ii) regulation of autophagy, glycosylphosphatidylinositol (GPI) anchor biosynthesis; (iii) neuroactive ligand-receptor interaction, long term depression and (iv) alpha linolenic acid metabolism, linoleic acid metabolism.

The resulting biological pathway analysis was consistent with prior studies. For example, the cytokine-cytokine receptor interaction pathway was reported to be the most enriched KEGG pathway associated with survival in GBM, using a network-based method (Jiang *et al.*, 2015). Another study reported that a panel of 18 cytokines discriminated GBM patients from healthy individuals, and the cytokine-cytokine receptor interaction and JAK-STAT pathways were the most enriched in pathway analysis (Nijaguna *et al.*, 2015). In particular, two cytokines (IL17 and IL4) were independently found to be good prognostic indicators. Arimappamagan *et al.* found 76 differentially expressed genes between low and high risk groups in GBM, and showed that these 76 genes were enriched in three KEGG pathways, including the cytokine-cytokine receptor interaction, NOD-like receptor signaling pathway and chemokine signaling pathway (Arimappamagan *et al.*, 2013), which are related to the inflammatory and immune response pathways. It has been found that GBM tumors secrete chemokines that are indirectly involved in angiogenesis by activating stromal cells, and can promote tumor growth and progression (Somasundaram and Herlyn, 2009). Remarkably, all three of these pathways were included in the first hot spot, resulting from the Grad-CAM procedure. Further, Tong *et al.* identified two modules from a protein-protein interaction network that were associated with survival in GBM, and showed that genes involved in the two network modules were mainly associated with the chemokine signaling pathway, neuroactive ligand-receptor interaction, ECM-receptor interaction and focal adhesion (Tong *et al.*, 2018). Moreover, some molecules in the ECM-receptor interaction pathway were shown to be associated with Temozolomide (TMZ) resistance (Zeng *et al.*, 2017). Consequently, these pathways are likely to be linked to prognosis in GBM.

The second hot spot included the neuroactive ligand receptor interaction and long-term depression pathways, both of which previously were not linked to GBM. However, Liu *et al.* demonstrated that some genes implicated in major depressive disorder were enriched with the neuroactive ligand receptor interaction pathway (Liu *et al.*, 2019). A study by Zhou et al. (2018) investigated differentially expressed genes between GBM samples and normal brain samples, and found that upregulated genes in a KEGG analysis were enriched in the neuroactive ligand-receptor interaction, cytokine-cytokine receptor interaction and JAK-STAT signaling pathways, implying that some common biological mechanisms exist between the neuroactive ligand receptor interaction and GBM-related pathways. Interestingly, the cytokine-cytokine receptor interaction and chemokine signaling pathways described above were found to be commonly enriched pathways in both major depressive disorder and GBM (Xie *et al.*, 2018). A report by Pal *et al.* (2018) also indicated that GBMs with the defective neuroactive ligand receptor interaction pathway had significantly worse prognoses.

The third hot spot included the pathways of GPI anchor biosynthesis and regulation of autophagy. Johnson *et al.* reported that GPI anchors are implicated in the tumorigenicity of GBM, and may indicate novel therapeutic alternatives (Johnson *et al.*, 2018). A study by Abdul Rahim *et al.* (2017) showed that ATG9A is essential for general GBM cell survival as a novel regulator of autophagy induction, and the inhibition of autophagy could be an effective therapy in GBM. However, targeting of autophagy in GBM treatment is still a matter of debate, and further in-depth investigations are needed to clearly understand the role of autophagy in GBM biology (Colella *et al.*, 2019).

The fourth hot spot included the linoleic acid metabolism and alpha linolenic acid metabolism, consisting of 29 and 19 genes, respectively, with 16 common genes. The linoleic acid metabolism and alpha linolenic acid are two essential fatty acids. Alpha linolenic acid is an isomer of gamma-linolenic acid. Recently, a paper reported that gamma-linolenic acid reduces the proliferation and migration of GBM cells and increases apoptosis, suggesting that gamma-linolenic acid has promising therapeutic potential in GBM (Miyake *et al.*, 2020).

An inherent limitation of our approach is that the identification of joint pathway hot spots requires contiguous alignment of the pathways in the pathway image. Pathway ordering currently maximizes the correlation of neighboring pathways. This could be extended in a future extension to include more neighboring configurations.

## 5 Conclusions

We have described PathCNN, a novel algorithm to build interpretable CNN models based on the concept of a ‘pathway image’, generated using multi-omics data. The model was able to predict long-term survival of GBM patients better than other common machine learning methods. Furthermore, the application of Grad-CAM directly on the pathway image enabled the identification of plausible pathways that affect survival in GBM. In summary, this study shows the potential of using CNNs on multi-omics data, along with Grad-CAM, to identify complex and non-linear biological correlates of disease aggressiveness.

## Funding

This research was funded in part through the National Institutes of Health/National Cancer Institute Cancer Center [P30 CA008748, R21 CA234752], Air Force Office of Sponsored Research (AFOSR) [FA9550-17-1-0435, FA9550-20-1-0029], Breast Cancer Research Foundation [BCRF-17-193] and the Ministry of Science [ICT 2019-0-01601].

*Conflict of Interest*: none declared.
